# A branch-and-Benders-cut algorithm for a bi-objective stochastic facility location problem

**DOI:** 10.1007/s00291-020-00616-7

**Published:** 2021-03-06

**Authors:** Sophie N. Parragh, Fabien Tricoire, Walter J. Gutjahr

**Affiliations:** 1 Institute of Production and Logistics Management, Johannes Kepler University Linz, Altenberger Straße 69, 4040 Linz, Austria; 2 Institute for Transport and Logistics Management, Vienna University of Economics and Business, Welthandelsplatz 1, 1020 Vienna, Austria; 3 Department of Statistics and Operations Research, University of Vienna, Oskar-Morgenstern-Platz 1, 1090 Vienna, Austria

**Keywords:** Bi-objective optimization, Stochastic optimization, Branch and bound, Benders decomposition, L-shaped method, Pareto efficiency

## Abstract

In many real-world optimization problems, more than one objective plays a role and input parameters are subject to uncertainty. In this paper, motivated by applications in disaster relief and public facility location, we model and solve a bi-objective stochastic facility location problem. The considered objectives are cost and covered demand, where the demand at the different population centers is uncertain but its probability distribution is known. The latter information is used to produce a set of scenarios. In order to solve the underlying optimization problem, we apply a Benders’ type decomposition approach which is known as the L-shaped method for stochastic programming and we embed it into a recently developed branch-and-bound framework for bi-objective integer optimization. We analyze and compare different cut generation schemes and we show how they affect lower bound set computations, so as to identify the best performing approach. Finally, we compare the branch-and-Benders-cut approach to a straight-forward branch-and-bound implementation based on the deterministic equivalent formulation.

## Introduction

Facility location problems play an important role in long-term public infrastructure planning. Prominent examples concern the location of fire departments, schools, post offices, or hospitals. They are not only relevant in public (or former public) infrastructure planning decisions in “regular” planning situations: they are also of concern in the context of emergency planning, e.g., relief goods distribution in the aftermath of a disaster or preparation for slow onset disasters such as droughts. In many of these contexts, accurate demand figures are not available; assumed demand values rely on estimates, while their actual realizations depend, e.g., on the severity of the slow onset disaster, the demographic population development in an urban district, etc. Since facility location decisions are usually long-term investments, the uncertainty involved in the demand figures should already be taken into account at the planning stage.

Another important issue is that facility location problems often involve several objectives. On the one hand, client-oriented objectives should be optimized. For example, in cases where it is not possible to satisfy the demand to 100 percent, the total covered demand should be as high as possible. On the other hand, cost considerations also play a role. This implies that decision makers face a trade-off between client-oriented and cost-oriented goals. Instead of combining these two usually conflicting measures into one objective function, it is advisable to elucidate their trade-off relationship. Such an approach provides valuable information to the involved stakeholders and allows for better informed decisions. Following this line of thought, in this paper, we model a bi-objective stochastic facility location problem that considers cost and coverage as two competing but concurrently analyzed objectives. Our concept of analysis is that of Pareto efficiency. Furthermore, we incorporate stochastic information on possible realizations of the considered demand figures in the form of scenarios sampled from probability distributions.

Motivated by recent advances in exact methods for multi-objective integer programming, we solve this problem by combining a recently developed bi-objective branch-and-bound algorithm (Parragh and Tricoire 2019) with the L-shaped method (Van Slyke and Wets 1969), which applies Benders decomposition (Benders 1962) to two-stage stochastic programming problems. We evaluate several enhancements, such as partial decomposition, and we compare the resulting approach to using a deterministic equivalent formulation within the same branch-and-bound framework.

This paper is organized as follows. In Sect. 2, we give a short overview of related work in the field of bi-objective (stochastic) facility location. In Sect. 3, we define the bi-objective stochastic facility location problem (BOSFLP) that is subject to investigation in this paper and we discuss L-shaped-based decomposition approaches of the proposed model. In Sect. 4, we explain how we integrate the proposed decomposition schemes into the bi-objective branch-and-bound framework. A computational study comparing the different approaches is reported in Sect. 5, and Sect. 6 concludes the paper and provides directions for future research.

## Related work

Our problem is a stochastic extension of a bi-objective *maximal covering location problem* (MCLP). The MCLP has been introduced in Church and ReVelle (1974). It consists in finding locations for a set of *p* facilities in such a way that a maximum population can be served within a pre-defined service distance. In this classical formulation, the number of opened facilities is a measure of cost, while the total population that can be served is a measure of demand coverage. Thus, cost occurs in a constraint, whereas the objective represents covered demand. It is natural to extend this problem to a *bi-objective* covering location problem (CLP) where both cost (to be minimized) and covered demand (to be maximized) are objectives. Indeed, bi-objective CLPs of this kind have been studied in several papers, see, e.g. Bhaskaran and Turnquist (1990), Harewood (2002), Villegas et al. (2006), or Gutjahr and Dzubur (2016a); for further articles, we refer to Farahani et al. (2010).

Another strand of literature relevant in the present context addresses bi-objective *covering tour problems* (CTPs). One of the oldest CTP models, the maximal covering tour problem (MCTP) introduced by Current and Schilling (1994) is actually a bi-objective problem. A fixed number of nodes have to be selected out of the nodes of a given transportation network; then a tour visiting these selected nodes must be determined. The objectives are minimization of the total tour length (a measure of cost) and maximization of the total demand that is satisfied within some pre-specified distance from a visited node (a measure of demand coverage).

Other multi-objective CTP formulations can be found in the following papers: Jozefowiez et al. (2007) deal with a bi-objective CTP where the first objective is, as in the original problem, the minimization of the total tour length while the second objective function of the MCTP is replaced by minimizing the largest distance between a node of some given set and the nearest visited node. Doerner et al. (2007) develop a three-objective CTP model for mobile health care units in a developing country. Nolz et al. (2010) study a multi-objective CTP addressing the problem of delivery of drinking water to the affected population in a post-disaster situation.

Most importantly for the present work, Tricoire et al. (2012) generalize the bi-objective CTP to the stochastic case by assuming uncertainty on demand. The aim is to support the choice of distribution centers (DCs) for relief commodities and of delivery tours supplying the DCs from a central depot. Demand in population nodes is assumed as uncertain and modeled stochastically. DCs have fixed given capacities, as well as vehicles. The model considers two objective functions: The first objective is cost (more precisely, the sum of opening costs for DCs and of transportation costs), and the second is expected uncovered demand. Contrary to basic covering tour models supposing a fixed distance threshold, uncovered demand is defined by the more general model assumption that the percentage of individuals who are able and willing to go to the nearest DC can be represented by a nonincreasing function of the distance to this DC. Both the demand of those individuals who stay at home and the demand of those individuals who are not supplied in a DC because of DC capacity and/or vehicle capacity limits contribute to the total uncovered demand. Because of the uncertainty on the actual demand, total uncovered demand is a random variable, the expected value of which defines the second objective function to be minimized.

The problem investigated in the present paper generalizes the bi-objective CLP to a stochastic bi-objective problem by considering demand as uncertain and modelling it by a probability distribution, in an analogous way as in Tricoire et al. (2012). Alternatively, the investigated problem can also be derived from a CTP by omitting the routing decisions and generalizing the resulting bi-objective location problem again to the stochastic case. From the viewpoint of the latter consideration, the current model can be seen as related to the special case of the problem of Tricoire et al. (2012) obtained by neglecting routing costs. However, the current model builds on refined assumptions concerning the decision structure of the two-stage stochastic program which makes the second-stage optimization problem nontrivial, contrary to Tricoire et al. (2012) where the second-stage optimization problem can be solved by elementary calculations. Thus, the main novelty of the proposed bi-objective location problem compared to existing models is that it defines a covering location model containing a proper two-step stochastic program (which is not reducible to a single stage) within a bi-objective optimization frame based on the determination of Pareto-optimal sets.

Multi-objective stochastic optimization (MOSO) problems, though of eminent importance for diverse practical applications, are investigated in a more limited number of publications, compared to the vast amount of literature both on multi-objective optimization and on stochastic optimization; for surveys on MOSO, we refer the reader to Caballero et al. (2004), Abdelaziz (2012), and Gutjahr and Pichler (2016c). Of special relevance for our present work are multi-objective *two-stage stochastic programming* models where the “multicriteria” solution concept is that of the determination of *Pareto-efficient solutions*, and where the first-stage decision contains *integer* decision variables. Most papers in this area assume that one of the two objectives only depends on the first-stage decision, whereas the other objective depends on the decisions in both stages. This holds also for Tricoire et al. (2012). Let us give three other examples: Fonseca et al. (2010) present a two-stage stochastic bi-objective mixed integer program for reverse logistics, with strategic decisions on location and function of diverse collection and recovery centers in the first stage, and tactical decisions on the flow of disposal from clients to centers or between centers in the second stage. The first objective is expected total cost, which depends both on the first-stage and second-stage decision, whereas the second objective, the obnoxious effect on the environment, only depends on the first-stage decision. Stochasticity is associated with waste generation and with transportation costs. Cardona-Valdés et al. (2011) deal with decisions on the location of DCs and on transportation flows in a two-echelon production distribution network. Uncertainty holds with respect to the demand. The first objective represents expected costs, whereas the second objective expresses the sum of the maximum lead times from plants to customers. The authors model the random distribution by scenarios and solve the two-stage programming model by the L-shaped method, a technique that we will also use in our present work. Ghaderi and Burdett (2019), finally, present a two-stage stochastic model for strategically transporting and routing hazardous material. They consider two objective functions, where the first one represents fixed costs for transfer yard locations, and the second one captures exposure risk and transportation costs. Again, the first objective only depends on the strategic decision in the first stage. Basically, the authors follow a weighted-sum approach, but also Pareto-optimal solutions are reported and visualized.

Our problem has a structure similar to the models cited above: while the covered demand depends on the decisions in both stages, the cost objective is already determined by the first-stage decision. This allows the development of an efficient solution algorithm. Contrary to Tricoire et al. (2012), we will not apply an $\varepsilon$-constraint method for the determination of Pareto-efficient solutions, but use instead a more recent method developed in Parragh and Tricoire (2019).

There is a body of literature on two-stage humanitarian logistics with location decisions in the first stage and demand satisfaction in the second stage; they differ on how they handle and combine the objectives on cost and coverage. Balcik and Beamon (2008) consider the maximisation of coverage, under budget constraints. Rawls and Turnquist (2010) combine all considered costs (facility location and size, commodity acquisition, stocking decision, shipment, inventory) in a weighted sum, together with a penalty cost for unused material. Alem et al. (2016) consider four different models, which correspond to four different ways to handle uncertainty. In all four models the budget is a constraint. The first model is similar to that of Rawls and Turnquist (2010). Their second model aims to obtain the best worst-case deviation from optimality across all considered scenarios, while their third and fourth model consider a weighted sum of costs plus a weighted measure of risk. Tofighi et al. (2016) use an interactive method to elicit preference from a decision maker, considering a number of measures of cost. However the uncovered demand is aggregated with inventory cost in one objective. A tri-objective second-stage problem is tackled using the weighted augmented $\varepsilon$-constraint method. Interestingly, none of these methods formally investigate the tradeoff between cost and coverage in a multi-objective fashion: either they focus on coverage as the single objective, or they aggregate coverage as another form of cost and add a weight (or penalty) to it.

Finally, let us also mention that the humanitarian logistics literature, which tackles facility location problems under high uncertainty and multiple objectives, also has been relatively prolific with regards to multi-objective stochastic optimization models and corresponding solution techniques (cf. Gutjahr and Nolz (2016b)). Let us give two examples of papers using both Pareto optimization and two-stage stochastic programs. Khorsi et al. (2013) propose a bi-objective model with objectives “weighted unsatisfied demand” and “expected cost”. Discrete scenarios from a set of possible disaster situations are applied to represent uncertainty. The $\varepsilon$-constraint method is used to solve the model. Rath et al. (2016) deal with the uncertain accessibility of transportation links and develop a two-stage stochastic programming model where in the first stage decisions on the locations of distribution centers have to be made, and in the second stage (based on current information on road availability) the transportation flows have to be organized. Objective functions are expected total cost and expected covered demand. The structure of the two-stage stochastic program is different from that in the current paper insofar as both objectives depend on both first-stage and second-stage decision variables, which requires specific (and computationally less efficient) solution techniques. For general information on humanitarian logistics, the reader is referred to the standard textbook by Tomasini and Van Wassenhove (2009). Two-stage stochastic programing approaches to this field (in a single-objective context) are reviewed in Grass and Fischer (2016). A good recent example for the application of Benders decomposition to a stochastic model for humanitarian relief network design is Elçi and Noyan (2018).

For an overview on facility location in general, we refer the reader to Hamacher and Drezner (2002). Standard textbooks on multi-objective optimization and on stochastic programming are Ehrgott (2005) and Birge and Louveaux (2011), respectively.

## Problem definition and decomposition

In the bi-objective stochastic facility location problem (BOSFLP) considered in this article, the demand at each node $i \in V$ is uncertain. By the random variable $W_i$, we denote the demand at node *i*. At each node *j*, a facility may be built. A facility at node *j* has a capacity $\gamma _j$ and operating costs $c_j$. Furthermore, facilities that are farther than a certain maximum distance $d_{max}$ from a demand point cannot cover it. In order to take this aspect into account, we consider the set $A = \{(i,j)| i,j \in V, d_{ij} \le d_{max} \}$ of possible assignments (*i*, *j*), where $d_{ij}$ denotes the distance of demand node *i* from a potential facility at node *j*. The two considered goals are to minimize the total costs for operating facilities and to maximize the expected covered demand. We transform the second objective into a minimization one by multiplying it by $-1$. We assume further that the facility opening decision has to be taken ”here-and-now” while the coverage of demand points by facilities can be decided once the demand is realized. These decisions are ”wait-and-see” decisions. The resulting two-stage decision problem can be approached using two-stage stochastic programming. The optimal solution value of the second-stage problem under first-stage decisions *z* and random variable $\xi$ is denoted $Q(z, \xi )$. A table giving an overview of the employed notation throughout the paper is given in Appendix A. We note that the demand at a node can be covered by a facility if that facility is open in the first stage, and as much as the capacity at that facility allows it. Partial coverage and split coverage are both possible, so for instance 60% of the demand of a population node can be covered by an open facility while 30% of that same demand is covered by another facility and 10% remain uncovered.

Using the following decision variables,

$$\begin{aligned} z_j =&{\left\{ \begin{array}{ll} 1,\text { if a facility is built at node }j\text { and } \\ 0,\text { otherwise.} \end{array}\right. }\\&\quad y_{ij} \text { demand of population node } i\text { that is covered by facility }j,\\&\quad u_j \text { total demand covered by the facility at }j. \end{aligned}$$

we formulate the BOSFLP as a two-stage stochastic program:123Second stage:

4 $$\begin{aligned} Q(z,\xi )&= \min _{u} \left( - \sum _{j \in V} u_j\right) \end{aligned}$$

subject to:5678910Objective function (1) minimizes the total facility opening costs. Objective function (2) maximizes the expected covered demand. In order to obtain two minimization objectives, objective (2) has been multiplied by $(-1)$. The first stage model only comprises one set of constraints which require that all $z_j$ variables may only take values 0 or 1. The second stage model consists of the objective function given in (4), representing the negative value of the total covered demand, and a number of constraints which determine the maximum possible coverage given a first stage solution. Constraints (5) link coverage variables $u_j$ with assignment variables $y_{ij}$: the covered demand at node *j* cannot be larger than the actual demand assigned to this node. Constraints (6) make sure that the capacity of facility *j* is not exceeded. Constraints (7) guarantee that a demand node can only be assigned to a facility if the respective facility is open. Finally, constraints (8) ensure that any part of the demand at *i* is only covered at most once. The variables $z_j$ are first-stage decision variables whereas the variables $y_{ij}$ are second-stage decision variables, i.e., the latter variables depend on the realizations of the demand values; that is, $y_{ij}=y_{ij}(\xi )$. Similarly, $u_j=u_j(\xi )$ and $W_i=W_i(\xi )$.

It should be noted that in our model, the decision maker is assumed as risk-neutral with respect to the covered demand, which implies that she only considers its expected value as a quantification of the second objective function.

Since the true demand distribution is too complex to be handled computationally within the optimization model, we resort to a scenario-based approach. The purpose of such an approach is to approximate the considered probability distribution by a discrete distribution on a (not too large) set of specific *scenarios*. As described in detail in Shapiro and Philpott (2007), a possible way to identify scenarios is *sample average approximation* (SAA); this method has also been applied in Ghaderi and Burdett (2019). SAA consists in generating a sample *N* of independent random realizations from the given distribution by Monte-Carlo simulation. Each realization can then be conceived as a scenario with assigned probability 1/|*N*|, and the expected value turns into an average over scenarios. Using an additional index $\nu$ to denote a given scenario for the variables of the second stage problem, we obtain the following expanded model:

11 $$\begin{aligned} \min f_1&= \sum _{j \in V} c_j z_{j} \end{aligned}$$

12 $$\begin{aligned} \min f_2&= - \frac{1}{|N|} \sum _{\nu \in N} \sum _{j \in V} u_j^{\nu } \end{aligned}$$

subject to:13141516171819Our aim is the determination of the set of Pareto-optimal solutions of the problem above, i.e., of all solutions that cannot be improved in one of the two objectives without a deterioration in the other objective.

Instead of considering a set of scenarios with equal probabilities $p_\nu = 1/|N|$, the model above can also be generalized to arbitrary probabilities $p_\nu > 0$ with $\sum _{\nu \in N} p_\nu = 1$ in a straightforward way.

Since we will solve the BOSFLP by means of a bi-objective branch-and-bound algorithm, we have to solve the linear relaxation of model (11)–(19) to compute lower bounds (see Sect. 4). For efficiently solving the linear relaxation, especially with a large number of scenarios, the well known L-shaped method, as introduced by Van Slyke and Wets (1969), is used. In the L-shaped method, the problem is decomposed into a so-called master problem (incorporating the first stage variables and constraints) and one subproblem per scenario. The information from the subproblems is included into the master problem by means of an additional variable reflecting the value of the second objective and by cutting planes, which are derived from iteratively solving the master problem and each of the scenario subproblems. In the case where the optimal solution to the master problem does not result in a feasible solution to the subproblem(s), a feasibility cut has to be added to the master. In the case where the expected value of the second objective is underestimated, an optimality cut is added to the master problem. If no additional cut has to be added, the procedure terminates and the optimal solution to the linear relaxation has been found. In the case of complete recourse, as is the case for our problem, only optimality cuts have to be added: a solution to the first stage problem will always allow a feasible solution to the second stage problem. (This holds for our problem, since no matter which facility opening decision is taken, setting all second stage variables, $y_{ij}^{\nu }$ and $u_j^{\nu }$, to zero produces a feasible solution, see constraints (14) and (15).) For an example of the L-shaped method, we refer to Birge and Louveaux (2011).

In the following, we shall specify:

- the master problem required by the L-shaped method [(20)–(23)],
- a linear program performing the optimality test for the current solution of the L-shaped algorithm [(24)–(30)],
- the dual of the latter program, which allows the derivation of optimality cuts [(31)–(37)], and
- an alternative (scenario-based instead of iteration-based) master problem variant for optimality cut generation [(41)–(44)].

Using variable $\theta$ to represent the second stage objective, we obtain the following master linear program (LP):

20 $$\begin{aligned} \min f_1 =&\sum _{j \in V} c_j z_j \end{aligned}$$

21 $$\begin{aligned} \min f_2 =&\, \theta \end{aligned}$$

subject to:2223where $- \sum \limits _{i \in V} \max \limits _{\nu \in N} \{ W^{\nu }_i \}$ provides a valid bound on $\theta$, since $Q(z,\xi ) = \min \limits _{u} \left( - \sum \limits _{j \in V} u_j\right) \ge - \sum \limits _{i \in V} \max \limits _{\nu \in N} \{ W^{\nu }_i \}$.

How the bi-objective nature of the problem is addressed is discussed in detail in Sect. 4. It requires the computation of lower bound sets which rely on iteratively solving single objective weighted sum problems. In that context and for a given set of weights, to determine if the obtained solution to the master weighted-sum LP is optimal, we check if optimality cuts have to be added. We denote by $z_j^l$ and $\theta ^l$ the variable values obtained from solving the master LP in iteration *l*, and we solve for each $\nu \in N$ the following model:

24 $$\begin{aligned} \min \left( - \sum _{j \in V} u_j^{\nu }\right) \end{aligned}$$

subject to:252627282930Let $\mathcal {Q}(z) = {\mathbf {E}}_{\xi }Q(z,\xi )$. If $\mathcal {Q}(z^l) \le \theta ^l$, we terminate: optimality has been reached. Otherwise, we generate an optimality cut. Optimality cuts rely on dual information. To write the dual of the above model (24)–(30), we denote by $\lambda _j$ the dual variables of constraints (25), by $\pi _j$ the dual variables of constraints (26), by $\sigma _{ij}$ the dual variables of constraints (27), and by $\delta _i$ the dual variables of constraints (28):

31 $$\begin{aligned} \max \left( - \sum _{j \in V} \pi _j \gamma _j z_j^l - \sum _{(i,j) \in A} \sigma _{ij} W_{i} z_j^l - \sum _{i \in V} W_{i} \delta _i\right) \end{aligned}$$

subject to:323334353637We denote by $\pi ^{\nu ,l}_j = \pi _j(\xi ^{\nu },z^l)$, $\sigma ^{\nu ,l}_{ij} = \sigma _{ij}(\xi ^{\nu },z^l)$, and $\delta ^{\nu ,l}_i = \delta _i(\xi ^{\nu },z^l)$ the dual variable values for a given scenario $\nu$, and by $Q(z^l, \xi ^{\nu }) = - \sum _{j \in V} \pi _j^{\nu ,l}\gamma _j z_j^l - \sum _{(i,j) \in A} \sigma _{ij}^{\nu ,l} W_{i}^{\nu } z_j^l - \sum _{i \in V} W_{i}^{\nu } \delta _i^{\nu ,l}$ the objective function value. Then, the optimality cut for scenario $\nu$ is of the following form (Van Slyke and Wets 1969):

38 $$\begin{aligned} Q(z,\xi ^{\nu })&\ge Q(z^l,\xi ^{\nu }) +\Bigg ( \Big [ - \sum _{j \in V} \pi _j^{\nu ,l} \gamma _j z_j - \sum _{(i,j) \in A} \sigma _{ij}^{\nu ,l} W_{i}^{\nu } z_j - \sum _{i \in V} \delta _i^{\nu ,l} W_{i}^{\nu } \Big ] \nonumber \\&\quad - \Big [ - \sum _{j \in V} \pi _j^{\nu ,l} \gamma _j z_j^l - \sum _{(i,j) \in A} \sigma _{ij}^{\nu ,l} W_{i}^{\nu } z_j^l - \sum _{i \in V} \delta _i^{\nu ,l} W_{i}^{\nu } \Big ] \Bigg ) \end{aligned}$$

Rearranging the terms, we obtain

39 $$\begin{aligned} Q(z,\xi ^{\nu }) \ge Q(z^l,\xi ^{\nu }) + \left( \sum _{j \in V} \pi _j^{\nu ,l} \gamma _j (z_j^l - z_j) + \sum _{(i,j) \in A} \sigma _{ij}^{\nu ,l} W_{i}^{\nu } (z_j^l - z_j) \right) \end{aligned}$$

Finally, combining over all scenarios, we obtain the optimality cut for the expected second stage objective function (with $\bar{\pi }_j^{l} = 1/|N| \sum _{\nu } \pi _j^{\nu ,l}$ and $\bar{\sigma }_{ij}^{l} =1/|N| \sum _{\nu } \sigma _{ij}^{\nu ,l} W_{i}^{\nu }$):

40 $$\begin{aligned} \theta \ge \mathcal {Q}(z^l) + \left( \sum _{j \in V} \bar{\pi }_j^{l} \gamma _j (z_j^l - z_j) + \sum _{(i,j) \in A} \bar{\sigma }_{ij}^{l} (z_j^l - z_j) \right) \end{aligned}$$

Alternatively, instead of adding one optimality cut per iteration, we can also add one cut per scenario. In order to do so, a separate variable $\theta ^{\nu }$ for each scenario $\nu$ has to be used, resulting in the following master LP:

41 $$\begin{aligned} \min f_1 =&\sum _{j \in V} c_j z_j \end{aligned}$$

42 $$\begin{aligned} \min f_2 =&\frac{1}{N} \sum _{\nu \in N} \theta ^{\nu } \end{aligned}$$

subject to:4344Then, we check each subproblem and add a cut of the form (39) in the case where $\theta ^{\nu ,l} < Q(z^l,\xi ^{\nu })$. In the case where all scenarios are checked and no additional cut has to be added, optimality has been reached.

## Solution methods

In order to solve the BOSFLP, we integrate L-shaped based cut generation into the recently introduced bi-objective branch-and-bound framework of Parragh and Tricoire (2019). In what follows, we first describe the key ingredients of the branch-and-bound framework and thereafter how we combine it with the L-shaped method.

### Bi-objective branch-and-bound

Without loss of generality, we consider a bi-objective *minimization* problem. The bi-objective branch-and-bound (BIOBAB) algorithm of Parragh and Tricoire (2019) generalizes the single-objective concept of branch-and-bound to two objectives. It is similar in structure to the well-known branch-and-bound algorithm for single-objective optimization (Land and Doig 1960): a search tree is explored and at each of its nodes bounding is performed, then branching (splitting the search space into subsets) as well if necessary, and eventually, subtrees are discarded if it is seen from the bounds that they cannot contain the optimal solution anymore. However, where single-objective branch-and-bound considers scalar upper- and lower-bound values, BIOBAB uses lower and upper bound *sets*. Special care is thus required for bounding and branching procedures, and *filtering* is a pre-requisite to branching. The general structure of BIOBAB is outlined in Algorithm 1. In the following, we first introduce the notion of lower and upper bound set. Thereafter, we explain the main loop of the algorithm in more detail and we describe several enhancements.

#### Lower and upper bound sets

During the execution of the BIOBAB algorithm, instead of single numerical values, upper and lower bound sets are computed. These rely on the notion of *bound set* introduced by Ehrgott and Gandibleux (2006). A subset *L* of the bi-dimensional objective space is called a lower bound set of the feasible set *Y* in objective space if $\forall v \in Y \exists x \in L: v \ge x$, where $v \ge x$ iff $v_p \ge x_p (p=1, 2)$. Starting from the root node, at each node of the branch-and-bound tree, a lower bound (LB) set is calculated. The special LB set we use corresponds to a portion of the boundary of the convex hull of the feasible set for the current node LP in objective space. This boundary can be described by its corner points which can be efficiently computed by means of an algorithm that is similar to that of Aneja and Nair (1979). This algorithm consists in solving a series of single-objective weighted-sum problems by systematically enumerating a finite set of weight combinations. For each weight combination we solve the linear relaxation of the weighted-sum problem, i.e., using objective $w_1 f_1 + w_2 f_2$, where $w_1$ and $w_2$ are the current weights; and as explained in Sect. 4.2.1, we will solve these weighted-sum problems by means of the L-shaped method. Considering objectives functions $f = (f_1, f_2)$, the *image* of a solution *x* in objective space is given by $f(x) = (f_1(x), f_2(x))$. The image of the solution to each relaxed weighted-sum problem gives a corner point of the boundary in objective space. In a first step, the algorithm computes the two extreme solutions, i.e., the best solution optimizing $f_1$ and the best solution optimizing $f_2$. Let *a* denote the point in objective space which is the image of the optimal solution for $f_1$ and *b* the point in objective space which is the image of the optimal solution for $f_2$. In order to obtain the best possible value for the respective other objective function, lexicographic minimization is used. The next step consists in identifying the weights for finding the next solution of the LB set. These weights are derived from *a* and *b*. Let $a_1$ and $a_2$ denote the coordinates of *a*, and $b_1$ and $b_2$ the coordinates of *b*. Then the weights to obtain the next solution of the LB set are $w_2 = b_1 - a_1$ and $w_1 = a_2 - b_2$. Let the image of this new solution in objective space be denoted by *c*. Then we look for additional solutions between *a* and *c* and between *c* and *b*, in the same way as before. The LB set generation algorithm is outlined in Appendix C. For further details we refer to Parragh and Tricoire (2019).

**Fig. 1:**
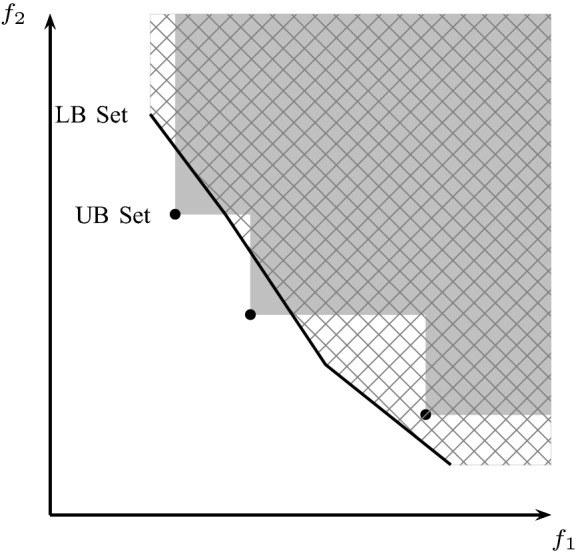
LB set (continuous) of a given branch-and-bound node, current UB set (dots), region dominated by LB set (grid pattern), region dominated by UB set (gray) in objective space. Non-dominated feasible solutions have to lie in the white grid-pattern area

The thus obtained LB set is then filtered using a set of known solutions, called the upper bound (UB) set. The UB set corresponds to all integer feasible solutions obtained during the search that have not been found to be dominated so far. In order to fathom a node, the whole LB set of this node must be dominated by the UB set. Note that the LB set and the UB set are conceptually different: while the former is a continuous set, the latter is a discrete set. This is illustrated in Fig. 1. The solid black line is the result of the LB set generation algorithm mentioned above. The space dominated by the LB set is visualized as the grid region bounded by the solid black line, and corresponds to all points that are dominated by a point on the solid black line. The UB set consists of three points, represented by three black dots. The space dominated by the three points, represented in a solid shade of gray, does not cover the entire LB set. Since portions of the LB set are not dominated by the UB set, the current node cannot be fathomed and branching is required.

#### Tree generation and branching rules

Algorithm 1 shows the main loop of the BIOBAB algorithm. *C* is the collection of nodes of the branch-and-bound tree that remain to be investigated. Function *push*(*C*, *x*) adds branch-and-bound node *x* to *C* while function *pop*(*C*) retrieves the next node to be processed from *C*. A node in the branch-and-bound tree represents a set of branching decisions. Depending on the data structure employed for *C*, different tree exploration strategies can be obtained. We use the depth-first exploration strategy, which consists in always prioritising branching over backtracking; for more details, see, e.g. Knuth (1997). The algorithm can take a starting UB set as input in order to speed up the search. In our case, the UB set passed to the algorithm is empty and it is updated every time a new integer solution is found.
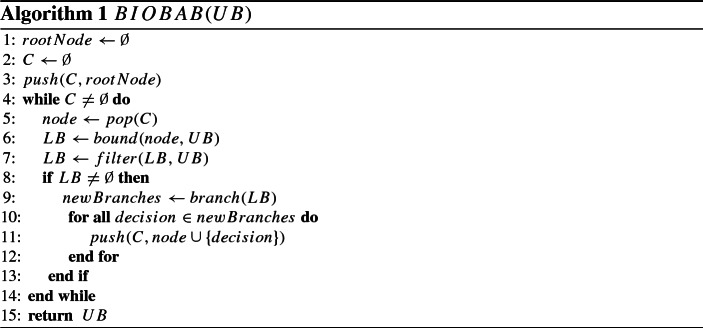


A key component of the BIOBAB algorithm of Parragh and Tricoire (2019) is a branching rule that works on the objective space, which is referred to as *objective space branching*. It allows to discard dominated regions of the search space even if a given node cannot be fathomed. The information whether or not objective space branching can be performed is obtained in the filtering step. Portions of the LB set that are dominated by some point of the UB set can be discarded, which is achieved via filtering. Whenever the current UB set allows to discard regions from the lower bound set and the resulting LB set is discontinuous, objective space branching in combination with variable branching is performed and each new branch (or node) corresponds to a different continuous subset of the discontinuous LB set. In the example depicted in Fig. 1, the filtering operation results in a discontinuous LB set consisting of three continuous subsets, which are the sections of the LB set that are not dominated by any point from the UB set, i.e., not in a gray shaded region. The parts of objective space that may yield new non-dominated points are the three regions that are both (i) covered by the grid pattern (i.e., dominated by the LB set) and (ii) not covered by the gray shade (i.e., not dominated by the UB set). Objective space branching allows to disregard all other regions.

Unless the LB set consists of a single integer solution, variable branching systematically happens, be it in combination with objective-space branching or by itself. The reason can be that a certain binary variable takes a fractional value, or that it takes different values in the different corner points defining the LB set. In any of these cases, branching on this variable involves creating two new nodes for the branch-and-bound tree, both having all branching decisions of the current node; additionally, one of the new nodes fixes this variable to 0 while the other fixes it to 1. The binary variable to branch on is selected based on information from all corner point solutions of the LB set: the variable that is fractional in the highest amount of corner points is selected for branching. Ties are broken by selecting the variable with lowest average distance to 0.5. If this is not enough, ties are broken by selecting the variable whose average value has the lowest distance to 0.5.

#### Enhanced objective space filtering

Another key component of the algorithm of Parragh and Tricoire (2019) are enhanced objective space filtering rules that rely on the observation that the objective values of integer solutions may only take certain values. In the simplest case, they are restricted to integer values. In Parragh and Tricoire (2019), only integer problems are addressed, where all coefficients in the objective function may only assume integer values. In this case, it is easy to observe that integer solutions may only assume integer objective values. In this paper, we solve a mixed integer program (the $y_{ij}$ and $u_j$ variables may assume fractional values). However, the continuous variables only appear in the second objective function. Thus, for the first objective function the same reasoning as in Parragh and Tricoire (2019) can be used. Our second objective function depends on continuous variables and, in addition, we divide by the number of scenarios to obtain the expected value. However, we can still exploit the ideas of Parragh and Tricoire (2019). The reasoning is as follows. Let us assume that all coefficients are integer valued (both in the constraints and in the objective functions). This implies that the capacities $\gamma _{j}$ of the distribution centers are integer valued as well as the demands at the demand nodes $W_i$. Then, it is easy to see that, in any optimal solution for a given scenario, at each distribution center, either the capacities are fully used (we maximize covered demand) or, in the case of excessive capacities, the entire demand of the reachable demand nodes is covered, resulting in an integer valued objective function. Now, fractional values can only be due to the term 1/|*N*|. Since this term is constant, we can simply multiply the second objective function by |*N*| to obtain integer valued results. If we do not want to do that, the constant term 1/|*N*| still allows us to know the granularity of the admissible values of the objective function; any region in the objective space which does not contain any admissible values can be removed from further consideration. This observation can be used to prune LB segments and to speed up the LB set computation procedure. For further details we refer to Parragh and Tricoire (2019).

### Lower bound set generation and integration with L-shaped method

Integrating the L-shaped method into BIOBAB mainly affects the lower bound set generation scheme. In what follows we first present the employed master program and then the employed cut generation strategies.

#### Master program

We now describe how we set up the master LP to use the L-shaped method for solving a weighted-sum problem inside of the lower bound computation scheme. Let $w_1$ and $w_2$ denote the weights as described in Sect. 4.1.1 and $\bar{f}_1$ and $\bar{f}_2$ the upper bounds on $f_1$ and $f_2$, respectively, we obtain the following generic master LP for the multi-cut version:

45 $$\begin{aligned} \min w_1 \sum _{j \in V} c_j z_j + w_2 \frac{1}{|N|} \sum _{\nu \in N} \theta ^{\nu } \end{aligned}$$

subject to:4647484950The optimality cuts as described in Sect. 3 are given in (47). Initially, the sets $S^{\nu }$ are empty. Whenever a cut for scenario $\nu$ is identified it is added to $S^{\nu }$; $Q^{\nu ,l} = Q(z^l,\xi ^{\nu })$ at iteration *l*. Similar notation is used to denote the dual variable values of iteration *l* and scenario $\nu$. Preliminary experiments indicated that, as expected, the multi-cut version performs better than the single cut version. For that reason, we focus on the multi-cut version. The model features bounds on both objectives to allow for easy updates in the case of objective space branching, which is realized by updating these bounds to discard dominated regions of the objective space. The weights in the objective function are determined by the algorithm of Aneja and Nair (1979). For each weight combination the L-shaped method is applied, i.e., optimality cuts (see Sect. 3) are generated as explained in the subsequent Section.

In order to strengthen the above master LP, we can use the following valid inequalities:

51 $$\begin{aligned} - \sum _{j \in V} z_j \gamma _j&\le \theta ^{\nu }\quad \forall \nu \in N \end{aligned}$$

They rely on the fact that the maximum coverage level is bounded by the total capacity of the number of opened facilities. By doing so, in the case where capacities are tight, we anticipate that fewer optimality cuts have to be added.

#### Optimality cut generation strategies

In the general case, the master program is solved, each scenario subproblem is solved and in the case where $\theta ^{\nu }$ is currently under-estimated an optimality cut is added and the master program is solved again. Optimality is attained if no additional cut has to be added. However, it is clearly not necessary to check all scenarios for valid cuts at each iteration: we can stop cut generation as soon as at least one cut has been found. In order to do so, several strategies can be envisaged and our preliminary experiments showed that the following strategy works reasonably well: at each call to the optimality cut generation routine, we do not start to check for cuts with the first scenario but we start with the scenario following the last scenario for which a cut was generated, i.e., if scenario 2 generated the last cut, in the next iteration we check scenario 3 first and iterate over the scenarios such that scenario 2 is the one checked last. This way, we always check first one of the scenarios that have not been checked for the longest time. In terms of cut management, we maintain a global cut pool and we keep all generated cuts in this pool.

In the single objective case it has been observed, e.g., by Adulyasak et al. (2015), that considerable performance gains are achieved if optimality cuts are only generated at incumbent solutions. Motivated by the success in the single objective domain, we transfer this idea to multi-objective optimization. We recall that we generate bound sets which are obtained by systematically solving a series of weighted-sum problems. In the current context, each weighted-sum problem corresponds to solving the above master program of the L-shaped method. In the case where fewer optimality cuts than necessary or even no optimality cuts are added, objective two is under-estimated and therefore we have a valid lower bound on the true value of objective two. This means that we do not really need to generate optimality cuts at each weighted-sum solution but we can restrict cut generation to those weighted-sum solutions which are integer feasible (mimicking the idea of adding cuts only at incumbent solutions). In the following we denote such a solution as an incumbent solution.

In the case where we find an incumbent solution, we do generate cuts then re-solve the modified LP with the same set of weights, in a loop, until one of two things happens:

1. the solution is not integer any more
2. the solution is integer and no more cuts can be generated

**Fig. 2:**
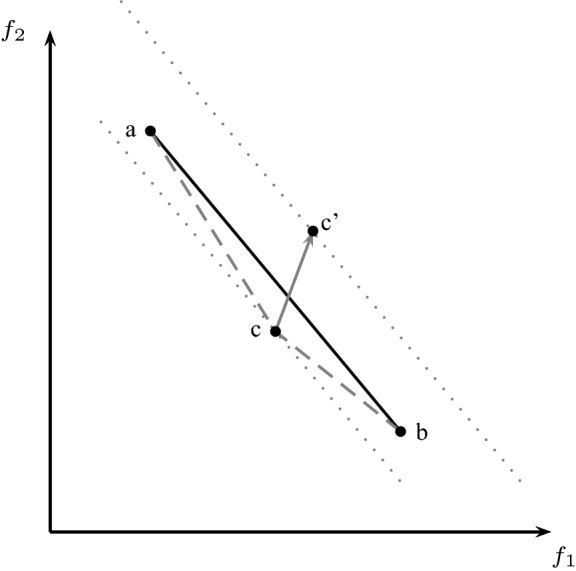
LB points *a* and *b*, new “incumbent” point *c*, through cut generation lifted to $c'$

The point thus obtained is then used as usual for LB set calculation purposes. Figure 2 depicts the situation where points *a* and *b* have been generated during LB set generation and the next step consists in investigating the segment between *a* and *b*. For this purpose the objective weights are set to $w_2 = b_1 - a_1$ and $w_1 = a_2 - b_2$ as described above and we obtain point *c*. Without cutting plane generation, the segments (*a*, *c*) and (*c*, *b*) would be investigated (dashed lines in the figure) by the LB set generation scheme. Now let us assume that *c* is an incumbent. This means that optimality cuts are generated and the cut generation loop results in a solution whose image in objective space is the point $c'$.

This point is *above* line (*a*, *b*). This is an issue, as the LB set algorithm only expects points below or on that line; this can lead to a non-convex LB set, because not every point in the convex hull boundary used the same cuts, i.e., the LP changed during the process. However the branch-and-bound algorithm relies on a convex LB set. Therefore, in such an eventuality, the new point is discarded for LB set calculation purpose, and the segment (*a*, *b*) is kept as valid (albeit not tight) LB segment. The LB segment is valid since the objective function value level curve of $c'$, depicted by a dotted line in Fig. 2, is a valid LB (set) (Stidsen et al. 2014).

#### Partial decomposition

Following Crainic et al. (2016), partial decomposition appears to be a viable option to obtain further speedups in the context of a Benders type algorithm. It refers to incorporating some of the scenarios into the master problem. Let $N^*$ denote the set of scenarios that are incorporated into the master LP, different strategies regarding which scenario should be part of $N^*$ can be envisaged. In the simplest case, the first scenario is put into $N^*$. After preliminary testing we decided to keep the scenario with lowest deviation from the average scenario, plus the *k* scenarios with highest deviation.

## Computational experiments

The previously described algorithms have been implemented using Python and Gurobi 8. The algorithms are run on a cluster with Xeon E5-2650v2 CPUs at 2.6 GHz. Each job is allocated 8 GB of memory and two hours of CPU effort. Multi-threading is disabled in Gurobi.

In what follows, we first give an overview of the considered benchmark instances. Thereafter, we compare the different methods and we discuss the obtained results.

### Benchmark instances

We use a set of 26 instances which are derived from real-world data from the region of Thiès in western Senegal (for further details on these data we refer to Tricoire et al. 2012). These instances feature between 9 and 29 vertices. Only 10 scenarios were used in Tricoire et al. (2012); we use 10, 50, 100, 200, 300, 400, 500, 600, 700, 800, 900 and 1000 scenarios for each instance. Scenarios are generated using a procedure similar to the one in Tricoire et al. (2012). Using more scenarios improves the quality of the approximation of the real situation but typically requires additional CPU effort.

Our benchmark serves the purpose to provide computational test instances, but for a practical application of the approach, the question from which stochastic model the scenarios are drawn obviously needs much attention. Let us mention that the considered disaster types are droughts which are basically natural disasters. This has the consequence that in principle, statistical data can be used for the estimation of their spatio-temporal occurrence distribution, and, in a second step, of demand distributions. For the sake of brevity, we do not address this aspect in the present paper. (As an example of statistical distribution estimation in a related model, we refer the reader to Doerner et al. (2009) where tsunami event distributions have been estimated from data.)

### Cut separation settings

We compare several settings. They all consist in solving the BOSFLP with BIOBAB, but differ in how the underlying single-objective weighted-sum stochastic optimization problems are tackled:

- *no decomposition*: instead of using the L-shaped method, the linear relaxation of the expanded model (11)–(19) is used in the lower bound computation scheme.
- *base*: base L-shaped method. No partial decomposition, no valid inequalities, cuts are systematically generated when they are violated.
- *partial decomposition*: the scenario with lowest deviation from average is built in the master problem, as well as the scenarios with highest deviation. Preliminary experiments indicated that building in more than 5 scenarios makes the initial model too large, i.e., any potential benefit is outweighed by the time it takes to solve the master problem. With 5 scenarios, additional preliminary experiments showed that keeping the scenario with lowest deviation from average plus the 4 scenarios with highest deviation from average performed better than other settings (such as 5 random scenarios or 5 scenarios with lowest deviation), therefore we use that setting.
- *valid inequalities*: the valid inequalities described at the end of Sect. 4.2.1 are added to the master problem.
- *incumbent cuts*: cuts are generated systematically at the root node of the tree search, then only on incumbent solutions, as described in Sect. 4.2.2.
- *incumbent cuts + valid inequalities*: Both strategies are used.

We first compare all settings in terms of CPU effort. Since there are 6 settings, 26 instances and 12 sample sizes, there are 1872 runs to compare. For that reason we present *performance profiles*. A performance profile is a chart that compares the performance of various algorithms (Dolan and Moré 2002). The performance of a setting for a given instance is the ratio of the CPU effort required with this setting for this instance over the best known CPU effort for the same instance. Lower values of performance indicate a better performance, and the best performance achievable is always 1. On a performance profile, performance is indicated on the *x*-axis while the *y*-axis indicates the ratio of instances solved with at least that level of performance by a certain setting. A curve reaching value 1 early, i.e., for a low value of the performance measure, indicates that 100% of instances are being solved for that level of performance or better. Therefore curves reaching value 1 early are desirable. If a certain setting does not converge in solving a given instance within the allotted CPU budget, then this setting does not provide a performance for that instance. This can be observed in the profile: the corresponding curve does not end up at value 1 but lower, indicating that for some instances the setting does not offer a performance.

**Fig. 3:**
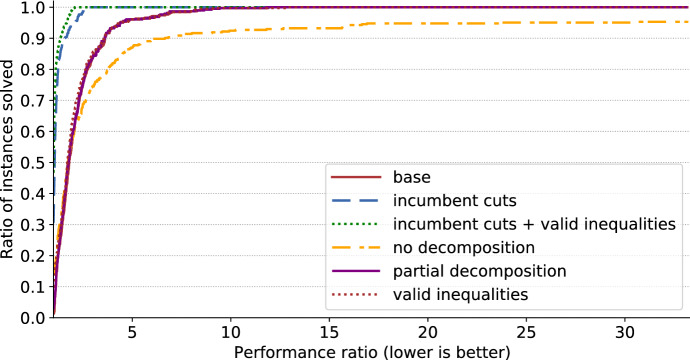
Performance profile: all settings on all test instances and all sample sizes

Figure 3 provides a comparison of the performance profiles of all six settings on all instances and all sample sizes. The only setting that does not always converge using the CPU budget is *no decomposition*, thus already emphasizing the need for decomposition. We can also see that among decomposition settings, some settings can be ten times faster than others. The two settings that only generate cuts on incumbent solutions appear to dominate the others.

**Fig. 4:**
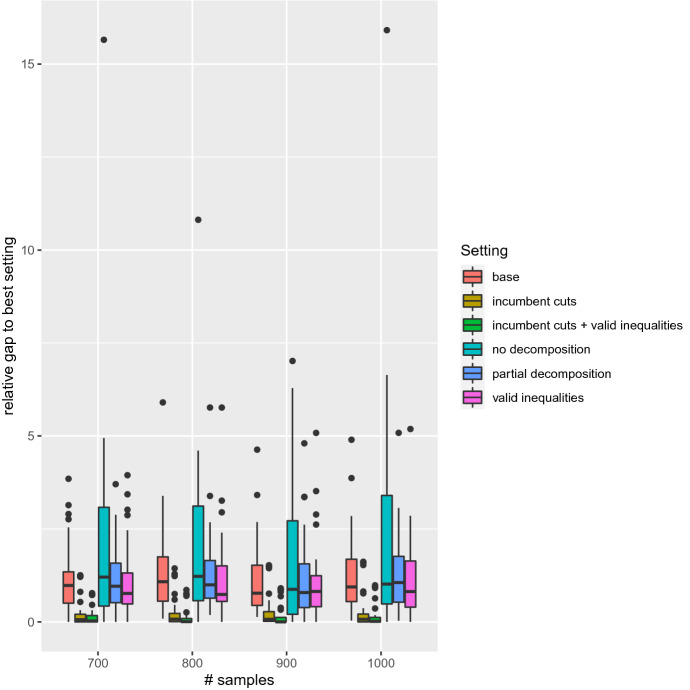
Box plot: relative gap to best setting. All settings on all test instances, large sample sizes only

For further insight, we now look at box plots for the same experimental data. This time the indicator is the relative gap to the best setting, i.e., if a given setting X is 50% slower than the fastest setting for a given instance, then the indicator value of setting X for that instance is 0.5. We use the *ggplot2* R package (Wickham 2016). Runs for which the algorithm does not converge are discarded. For the sake of readability, we only consider instances with at least 700 scenarios. This box plot is depicted in Fig. 4. We can now see that in certain cases, some methods are actually more than 15 times slower than the best method. It appears even more clearly that on large instances, which are the most interesting ones since they provide a better approximation of reality, settings generating cuts only for incumbent solutions perform better.

**Fig. 5:**
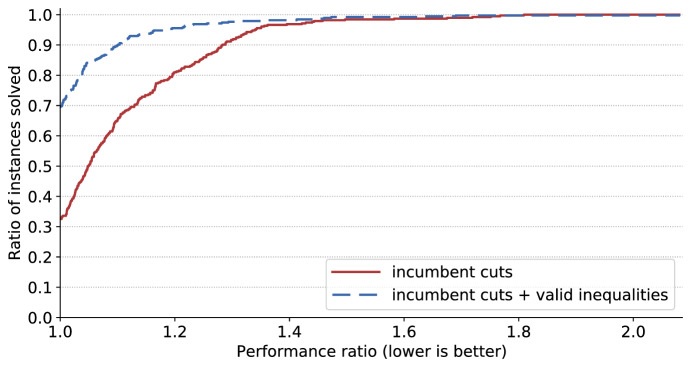
Performance profile: best settings on all test instances and all sample sizes

We now look at the two best settings only, in order to determine whether the valid inequalities provide any kind of significant improvement. For that purpose we first look at the performance profiles. They are depicted in Fig. 5. The setting that includes valid inequalities is not always the best, as indicated by the fact that it does not start at 1. However, its curve is way above the one from the setting without valid inequalities, indicating a better performance overall. In general, neither setting offers any very bad performance.

**Fig. 6:**
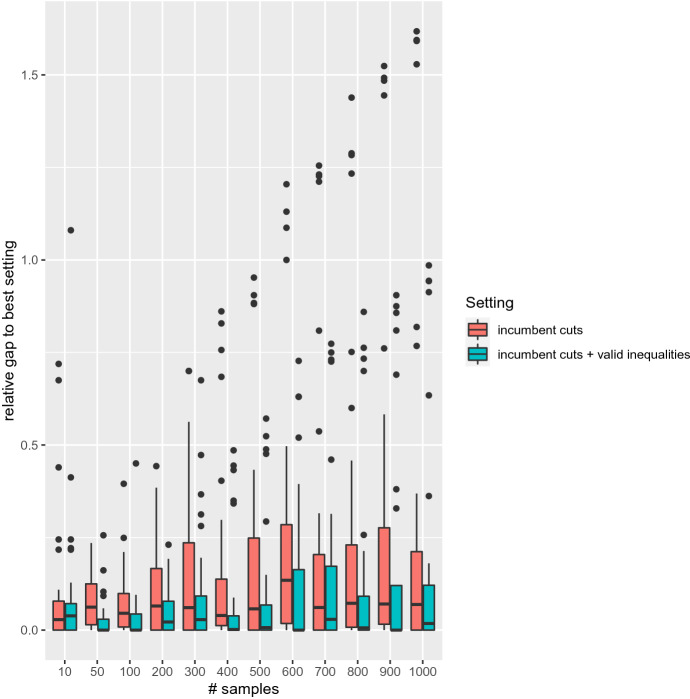
Box plot: relative gap to best setting. Best settings on all test instances and all sample sizes

We also provide a box plot for the two best settings in Fig. 6. As we can see the worst performance is below 2, meaning than no setting is ever twice as slow as the best known setting. This, together with prior graphics, indicates that generating cuts only at incumbent solutions is the main cause of good performance. However, there is a clear trend in favor of the setting that also includes valid inequalities, observed for all sample sizes but the smallest (10).

Based on these observations, it is clear that in our tests, the best setting is the one which both (i) only generates cuts at incumbent solutions and (ii) includes valid inequalities in the master problem, i.e., *incumbent cuts + valid inequalities*.

### Benchmark data

In order to facilitate future comparisons, we provide detailed results on each instance for the overall best setting, which is *incumbent cuts + valid inequalities*. These results can be found in Appendix B.

### Scenario reduction

As it is seen from the reported results, our approach is able to deal with a large number of scenarios. This is a nice property: improving solution algorithms in order to make larger number of scenarios computationally tractable has recently found attention also in the literature on stochastic models for humanitarian logistics. For example, Grass et al. (2020) propose an accelerated L-shaped method for solving two-stage stochastic programs in disaster management, showing for a hurricane preparedness model that the new algorithm is able to extend the number of scenarios from 15, as in Rawls and Turnquist (2010), to more than 100 scenarios. Grass et al. argue that scenario choices that reflect reality only insufficiently can essentially deteriorate the performance of the derived humanitarian operations, so that an extension of the scenario sets to cover a broad range of possible future events is highly desirable.

In addition to the already achieved efficiency of our algorithm, computation times for the considered application problem could be further reduced by the application of scenario-reduction techniques. The Monte-Carlo generation approach from Tricoire et al. (2012), on which we build, would lend itself especially well to *Importance Sampling* (see Birge and Louveaux (2011), ch. 10.2). This scenario-reduction technique has recently also been successfully applied in another disaster relief application, see Sang et al. (2018). The idea of Importance Sampling is to change the joint probability density $\varphi (w)$ of the uncertain parameters $W = (W_i)$ (in our case: the demands) from the realistic estimate to a modified density $\psi (w)$ that favors scenarios of specific interest for the objective function under consideration. In our case, this would be demand vectors with especially large demand values, such that our second objective function attains high values. Finding relatively cheap solutions even under these aggravated conditions is crucial for the optimization algorithm, whereas it is comparably easy to achieve under a low-demand regime. To compensate for the effect of the probability change, in the estimate of the objective function, the original weighted average (in our case the weights are identical) has to be replaced by weighing the terms additionally with the likelihood ratios $\varphi (w)/\psi (w)$. If the probability change is suitably chosen, the described procedure can reduce the variance of the objective function estimate and allows it in this way to get along with a smaller set of scenarios without deteriorating its representativeness. We suggest this issue as a topic for future research.

## Conclusions and outlook

We have defined a bi-objective facility location problem (BOSFLP), which considers both a deterministic objective (cost minimization) and a stochastic one (population coverage maximization), where the value of the second objective is evaluated by sample average approximation. The aim of the BOSFLP is to determine the set of efficient solutions using the Pareto approach, but aiming for good approximations with regard to the stochastic objective means considering large samples of random realizations, which makes using the expanded model directly impractical. We decomposed the original problem using Benders decomposition (L-shaped method) in a bi-objective branch-and-bound (BIOBAB) algorithm. This is, to the best of our knowledge, the first time that Benders decomposition has been integrated into a bi-objective branch-and-bound algorithm.

We also implemented several known improvements to the L-shaped method, and adapted them to the bi-objective context. Among all settings, we observed that generating Benders cuts only at the root node and at integer solutions speeds up the search considerably. The developed strategy for integrating cutting plane generation into the lower bound set algorithm generalizes to any type of cut and thus paves the way for the development of general purpose bi-objective branch-and-cut algorithms relying on bound sets. We also observed that valid inequalities on bounds for sample-dependent values of the stochastic objective bring a significant improvement. In both cases, experimental observations were significant enough to justify making these recommendations permanent, at least in the context of the BOSFLP.

Research perspectives include the incorporation of additional enhancements into the L-shaped method. Crainic et al. (2016) propose to use more sophisticated partial decomposition strategies, such as the clustering-mean strategy in which similar scenarios are clustered and a good representative from each cluster is incorporated into the master program, or the convex hull strategy, where scenarios that include other scenarios in their convex hull are integrated in the master program. Magnanti and Wong (1981) suggest improvements based on the notion of Pareto optimal cuts, a concept which has also been successfully employed by Adulyasak et al. (2015). The literature on the single-objective L-shaped method is abundant, and there are still lessons to be learnt in applying these techniques to the bi-objective context. Moreover, as illustrated by Tricoire et al. (2012), in the bi-objective context there is potential for improvements that rely on the interaction of the two objectives; one of our future goals is to develop bi-objective specific improvements for Benders decomposition techniques.

An important issue of future research is an extension of the chosen stochastic programming approach assuming a known probability distribution of the uncertain parameters to the consideration of *ambiguity*, i.e., uncertainty on the stochastic model itself (cf. Pflug and Wozabal 2007). In the application context of the present paper, this will be of particular interest for a treatment of man-made (or partially man-made) disasters.

## Notation

See Table 1.

**Table 1:** Notation

Sets and parameters	
*A*	Set of arcs (*i*, *j*)
$c_j$	Cost for operating a facility at node *j*
$d_{ij}$	Distance from node *i* to node *j*
$d_{max}$	Maximum distance for coverage
$\mathbf {E}(.)$	Expected value
$f_1$	Objective one
$f_2$	Objective two
*L*	Lower bound set
$S^{\nu }$	Set of optimality cuts
*N*	Set of scenarios (index $\nu$)
$Q(z, \xi )$	Objective value of second stage solution under first stage decisions *z* and random variable $\xi$
*V*	Set of nodes
$w_1, w_2$	Objective weights
$W_i^{(\nu )}$	Demand (random) at node *i* (in scenario $\nu$)
*Y*	Feasible set in objective space
$\gamma _j$	Capacity of facility at node *j*
$\xi$	Random variable

Decision variables	
$u_j^{(\nu )}$	Continuous decision variable indicating the total demand covered by facility *j* (in scenario $\nu$)
$y_{ij}^{(\nu )}$	Continuous decision variable giving the demand of population node *i* that is covered by facility *j* (in scenario $\nu$)
$z_j$	Binary decision variable indicating if a facility is built at node *j* (1) or not (0)
$\delta _i$	Dual variables of constraints (28)
$\lambda _j$	Dual variables of constraints (25)
$\pi _j$	Dual variables of constraints (26)
$\sigma _{ij}$	Dual variables of constraints (27)
$\theta ^{(\nu )}$	Variable used to represent the second stage objective (for scenario $\nu$)

## Benchmark results

We report benchmark results for the best setting, *incumbent cuts + valid inequalities*. Other settings may offer a better performance in some cases, but this is the setting that works best overall. Each table corresponds to a different sample size, and each table row provides indicators for one run. Indicators for each run are the number of vertices in the instance (*vertices*), the number of linear programs solved (*LPs*), the number of branch-and-bound nodes (*B&B nodes*), the number of cuts generated (*cuts*) and the CPU effort in seconds (*CPU*) (Tables 2, 3, 4, 5, 6, 7, 8, 9, 10, 11, 12, 13).

**Table 2:** Indicators for *incumbent cuts + valid inequalities* (10 samples)

Instance	Vertices	LPs	B&B nodes	Cuts	CPU (s)
Ndiakhene	9	11	1	35	0.19
Cherif_Lo	10	143	21	78	0.44
Thienaba	10	122	33	56	0.35
Ndieyene_Shirak	11	164	25	92	0.52
Notto_Gouye_Diama	11	34	7	95	0.3
Malicounda_Wolof	12	16	1	48	0.24
Mbayene	12	220	41	69	0.56
Pekesse	12	68	7	92	0.4
Thiadiaye	13	25	3	77	0.31
Thilmanka	14	25	1	60	0.31
Mont_Roland	15	71	9	110	0.54
Sandira	15	32	5	80	0.37
Koul	16	245	39	126	0.86
Meouane	17	137	31	125	0.61
Neugeniene	17	130	11	129	0.75
Ndiass	18	25	1	148	0.58
Pire_Goureye	18	243	31	143	1.0
Merina_Dakhar	19	116	19	150	0.86
Ngandiouf	19	763	167	72	1.71
Nguekhokh	19	63	3	156	0.77
Touba_Toul	20	511	105	183	1.46
Tassette	21	425	79	252	1.75
Diender_Guedj	22	1075	197	271	2.91
Ndiagagniao	24	4632	1165	254	7.26
Notto	28	6311	1591	262	10.6
Pout	29	5391	1513	321	8.91

**Table 3:** Indicators for *incumbent cuts + valid inequalities* (50 samples)

Instance	Vertices	LPs	B&B nodes	Cuts	CPU (s)
Ndiakhene	9	10	1	105	0.34
Cherif_Lo	10	166	17	288	1.47
Thienaba	10	125	27	250	1.17
Ndieyene_Shirak	11	443	83	279	2.32
Notto_Gouye_Diama	11	19	3	191	0.66
Malicounda_Wolof	12	16	1	115	0.51
Mbayene	12	267	45	238	2.01
Pekesse	12	250	31	329	2.22
Thiadiaye	13	31	3	167	0.85
Thilmanka	14	25	1	125	0.75
Mont_Roland	15	167	31	256	1.6
Sandira	15	67	17	179	1.3
Koul	16	205	31	296	2.69
Meouane	17	75	9	317	2.31
Neugeniene	17	115	11	362	2.78
Ndiass	18	170	39	359	2.46
Pire_Goureye	18	322	67	369	4.25
Merina_Dakhar	19	504	115	313	3.76
Ngandiouf	19	357	67	252	4.23
Nguekhokh	19	83	3	321	2.93
Touba_Toul	20	968	203	541	7.54
Tassette	21	349	51	481	6.16
Diender_Guedj	22	1131	231	678	9.62
Ndiagagniao	24	3549	901	802	20.21
Notto	28	10613	2733	928	55.29
Pout	29	4640	1205	721	24.47

**Table 4:** Indicators for *incumbent cuts + valid inequalities* (100 samples)

Instance	Vertices	LPs	B&B nodes	Cuts	CPU (s)
Ndiakhene	9	11	1	185	0.52
Cherif_Lo	10	166	15	557	3.11
Thienaba	10	125	27	462	2.21
Ndieyene_Shirak	11	306	63	601	4.34
Notto_Gouye_Diama	11	53	11	307	1.41
Malicounda_Wolof	12	16	1	250	0.9
Mbayene	12	216	31	367	3.18
Pekesse	12	110	13	626	2.96
Thiadiaye	13	46	7	370	1.89
Thilmanka	14	24	1	267	1.42
Mont_Roland	15	487	99	651	4.67
Sandira	15	37	5	311	2.01
Koul	16	228	37	678	5.76
Meouane	17	147	23	542	4.79
Neugeniene	17	232	31	597	6.12
Ndiass	18	213	49	700	6.2
Pire_Goureye	18	412	93	783	8.85
Merina_Dakhar	19	465	101	571	7.12
Ngandiouf	19	289	57	464	8.47
Nguekhokh	19	418	71	810	9.77
Touba_Toul	20	2040	437	1082	20.34
Tassette	21	179	19	834	9.15
Diender_Guedj	22	1062	219	1197	20.11
Ndiagagniao	24	3231	817	1454	39.72
Notto	28	15976	4073	1695	141.32
Pout	29	6290	1659	1486	62.84

**Table 5:** Indicators for *incumbent cuts + valid inequalities* (200 samples)

Instance	Vertices	LPs	B&B nodes	Cuts	CPU (s)
Ndiakhene	9	11	1	454	1.19
Cherif_Lo	10	191	17	1127	7.04
Thienaba	10	139	25	781	4.48
Ndieyene_Shirak	11	199	39	825	6.94
Notto_Gouye_Diama	11	20	3	580	2.37
Malicounda_Wolof	12	17	1	405	1.88
Mbayene	12	252	43	745	7.67
Pekesse	12	392	61	1401	9.73
Thiadiaye	13	33	3	537	3.26
Thilmanka	14	25	1	488	3.34
Mont_Roland	15	650	141	1471	12.47
Sandira	15	70	15	574	5.16
Koul	16	423	79	1445	14.95
Meouane	17	145	19	931	9.03
Neugeniene	17	242	33	1105	12.35
Ndiass	18	308	77	1180	13.19
Pire_Goureye	18	720	155	1618	21.44
Merina_Dakhar	19	560	127	1135	15.81
Ngandiouf	19	364	71	843	19.1
Nguekhokh	19	471	97	1473	18.5
Touba_Toul	20	1419	275	2316	40.58
Tassette	21	319	53	1567	23.5
Diender_Guedj	22	2232	463	2598	63.0
Ndiagagniao	24	4238	1125	1806	69.05
Notto	28	12136	3121	2686	217.35
Pout	29	5609	1469	2739	115.0

**Table 6:** Indicators for *incumbent cuts + valid inequalities* (300 samples)

Instance	Vertices	LPs	B&B nodes	Cuts	CPU (s)
Ndiakhene	9	11	1	630	1.62
Cherif_Lo	10	181	23	1385	10.43
Thienaba	10	172	35	1281	5.48
Ndieyene_Shirak	11	343	45	1579	14.72
Notto_Gouye_Diama	11	16	1	984	3.48
Malicounda_Wolof	12	17	1	595	2.78
Mbayene	12	211	31	981	9.13
Pekesse	12	295	43	1611	12.07
Thiadiaye	13	47	7	1025	6.16
Thilmanka	14	25	1	620	4.98
Mont_Roland	15	577	115	1683	17.02
Sandira	15	56	11	931	7.73
Koul	16	416	79	1745	20.66
Meouane	17	242	41	1419	16.26
Neugeniene	17	209	25	1573	20.51
Ndiass	18	511	113	2200	26.37
Pire_Goureye	18	396	65	1784	28.09
Merina_Dakhar	19	461	111	1626	25.53
Ngandiouf	19	342	61	1123	26.73
Nguekhokh	19	285	57	1868	23.96
Touba_Toul	20	1877	377	3125	69.57
Tassette	21	255	33	2104	32.76
Diender_Guedj	22	2077	417	3506	104.28
Ndiagagniao	24	5820	1409	4107	205.17
Notto	28	9836	2497	3624	302.43
Pout	29	6867	1829	4184	212.21

**Table 7:** Indicators for *incumbent cuts + valid inequalities* (400 samples)

Instance	Vertices	LPs	B&B nodes	Cuts	CPU (s)
Ndiakhene	9	11	1	720	2.12
Cherif_Lo	10	103	9	1832	11.16
Thienaba	10	145	29	1649	11.08
Ndieyene_Shirak	11	208	29	1968	14.97
Notto_Gouye_Diama	11	16	1	1055	4.35
Malicounda_Wolof	12	17	1	926	4.71
Mbayene	12	293	55	1415	17.67
Pekesse	12	278	27	2603	21.68
Thiadiaye	13	32	3	1319	7.89
Thilmanka	14	25	1	921	7.47
Mont_Roland	15	516	105	2108	23.31
Sandira	15	64	13	1266	11.55
Koul	16	391	77	2486	29.5
Meouane	17	208	35	1815	20.94
Neugeniene	17	165	23	2400	26.68
Ndiass	18	667	157	2731	39.02
Pire_Goureye	18	515	103	2665	39.09
Merina_Dakhar	19	698	163	2205	35.16
Ngandiouf	19	601	115	1633	45.38
Nguekhokh	19	420	109	2604	36.78
Touba_Toul	20	1752	347	5580	110.54
Tassette	21	290	37	2809	50.14
Diender_Guedj	22	1172	241	4265	99.05
Ndiagagniao	24	3502	835	4297	184.15
Notto	28	15150	3825	7627	724.75
Pout	29	7215	1903	6584	337.43

**Table 8:** Indicators for *incumbent cuts + valid inequalities* (500 samples)

Instance	Vertices	LPs	B&B nodes	Cuts	CPU (s)
Ndiakhene	9	11	1	870	2.84
Cherif_Lo	10	109	11	2375	16.8
Thienaba	10	215	41	1962	14.68
Ndieyene_Shirak	11	689	143	3782	35.66
Notto_Gouye_Diama	11	35	7	1325	7.6
Malicounda_Wolof	12	17	1	999	5.44
Mbayene	12	178	33	1561	16.44
Pekesse	12	386	53	3319	31.52
Thiadiaye	13	160	33	2400	14.45
Thilmanka	14	24	1	1265	8.8
Mont_Roland	15	632	131	3226	35.6
Sandira	15	66	13	1491	13.3
Koul	16	411	75	3569	49.5
Meouane	17	187	33	2325	28.24
Neugeniene	17	225	27	2836	40.15
Ndiass	18	465	139	2943	42.23
Pire_Goureye	18	707	151	3290	53.78
Merina_Dakhar	19	687	149	2490	41.37
Ngandiouf	19	746	159	1844	57.26
Nguekhokh	19	765	199	3464	55.49
Touba_Toul	20	1446	293	5629	125.24
Tassette	21	203	29	3100	46.08
Diender_Guedj	22	2549	545	6222	219.84
Ndiagagniao	24	3175	811	4359	216.4
Notto	28	10300	2627	7026	677.45
Pout	29	9699	2615	7750	531.69

**Table 9:** Indicators for *incumbent cuts + valid inequalities* (600 samples)

Instance	Vertices	LPs	B&B nodes	Cuts	CPU (s)
Ndiakhene	9	11	1	1000	3.22
Cherif_Lo	10	194	21	2697	26.34
Thienaba	10	243	59	2480	15.39
Ndieyene_Shirak	11	377	57	3911	40.69
Notto_Gouye_Diama	11	25	3	1627	7.89
Malicounda_Wolof	12	16	1	1200	6.12
Mbayene	12	286	59	1856	21.41
Pekesse	12	117	11	3329	24.19
Thiadiaye	13	46	7	1991	13.96
Thilmanka	14	25	1	1340	10.53
Mont_Roland	15	766	161	4389	56.04
Sandira	15	68	13	1811	13.83
Koul	16	370	69	4140	55.05
Meouane	17	269	47	2951	42.71
Neugeniene	17	170	17	3262	51.38
Ndiass	18	468	113	3462	53.77
Pire_Goureye	18	736	149	3864	81.04
Merina_Dakhar	19	630	141	3022	53.24
Ngandiouf	19	343	65	2549	68.44
Nguekhokh	19	893	225	4289	81.24
Touba_Toul	20	1410	237	7485	185.48
Tassette	21	359	55	3737	76.48
Diender_Guedj	22	1747	383	6754	197.8
Ndiagagniao	24	4169	1085	6062	348.91
Notto	28	10196	2545	7830	824.91
Pout	29	5767	1547	7242	417.66

**Table 10:** Indicators for *incumbent cuts + valid inequalities* (700 samples)

Instance	Vertices	LPs	B&B nodes	Cuts	CPU (s)
Ndiakhene	9	11	1	1416	4.94
Cherif_Lo	10	199	21	3102	30.55
Thienaba	10	191	39	2310	22.69
Ndieyene_Shirak	11	395	69	4471	44.67
Notto_Gouye_Diama	11	23	3	1804	7.97
Malicounda_Wolof	12	16	1	2105	9.5
Mbayene	12	192	25	2385	29.63
Pekesse	12	241	35	4197	37.8
Thiadiaye	13	49	7	2341	15.59
Thilmanka	14	24	1	1745	13.53
Mont_Roland	15	634	127	4459	55.65
Sandira	15	61	13	2272	20.31
Koul	16	472	93	4649	73.74
Meouane	17	148	23	3526	37.49
Neugeniene	17	338	59	3648	60.13
Ndiass	18	609	141	4299	82.22
Pire_Goureye	18	620	121	4307	81.37
Merina_Dakhar	19	1046	249	4454	88.32
Ngandiouf	19	418	73	2874	81.81
Nguekhokh	19	1112	281	5363	94.39
Touba_Toul	20	3078	677	7367	254.06
Tassette	21	365	61	4757	90.2
Diender_Guedj	22	1862	393	7655	271.29
Ndiagagniao	24	3690	937	6732	362.6
Notto	28	12753	3273	9053	1153.8
Pout	29	8741	2339	12372	892.39

**Table 11:** Indicators for *incumbent cuts + valid inequalities* (800 samples)

Instance	Vertices	LPs	B&B nodes	Cuts	CPU (s)
Ndiakhene	9	11	1	1390	4.67
Cherif_Lo	10	214	23	4416	37.43
Thienaba	10	182	37	2525	20.82
Ndieyene_Shirak	11	253	43	4035	36.19
Notto_Gouye_Diama	11	45	9	2249	12.34
Malicounda_Wolof	12	17	1	1615	8.8
Mbayene	12	219	37	2676	35.43
Pekesse	12	103	13	3857	29.22
Thiadiaye	13	49	7	2735	19.43
Thilmanka	14	24	1	1580	14.12
Mont_Roland	15	582	113	4445	58.15
Sandira	15	83	17	2110	24.33
Koul	16	472	91	5776	93.74
Meouane	17	237	39	3863	58.61
Neugeniene	17	258	39	3767	75.57
Ndiass	18	330	85	4427	66.73
Pire_Goureye	18	554	111	4735	80.23
Merina_Dakhar	19	357	73	3849	63.43
Ngandiouf	19	572	107	3332	103.31
Nguekhokh	19	784	223	5581	93.88
Touba_Toul	20	2121	427	8548	258.43
Tassette	21	297	39	5746	110.27
Diender_Guedj	22	2298	487	8687	351.12
Ndiagagniao	24	4547	1109	9431	548.11
Notto	28	14639	3699	11121	1522.24
Pout	29	5104	1275	10398	651.21

**Table 12:** Indicators for *incumbent cuts + valid inequalities* (900 samples)

Instance	Vertices	LPs	B&B nodes	Cuts	CPU (s)
Ndiakhene	9	11	1	1555	5.21
Cherif_Lo	10	117	11	3715	34.87
Thienaba	10	228	49	3486	30.81
Ndieyene_Shirak	11	385	67	4937	51.89
Notto_Gouye_Diama	11	63	13	2352	14.58
Malicounda_Wolof	12	17	1	2135	10.49
Mbayene	12	295	59	3287	42.85
Pekesse	12	315	45	4949	54.64
Thiadiaye	13	43	7	3120	22.09
Thilmanka	14	25	1	2040	17.46
Mont_Roland	15	906	201	7784	113.98
Sandira	15	77	17	2643	27.51
Koul	16	448	87	5799	102.2
Meouane	17	292	53	4488	76.73
Neugeniene	17	245	35	5181	85.7
Ndiass	18	554	129	6010	108.32
Pire_Goureye	18	446	81	6068	123.23
Merina_Dakhar	19	561	113	4709	107.37
Ngandiouf	19	474	87	3664	107.18
Nguekhokh	19	520	151	5168	89.1
Touba_Toul	20	2233	449	12664	380.63
Tassette	21	489	85	5656	134.4
Diender_Guedj	22	2848	617	11535	492.3
Ndiagagniao	24	4065	1009	10882	642.0
Notto	28	9397	2361	11463	1171.44
Pout	29	7902	2129	14670	1056.83

**Table 13:** Indicators for *incumbent cuts + valid inequalities* (1000 samples)

Instance	Vertices	LPs	B&B nodes	Cuts	CPU (s)
Ndiakhene	9	11	1	1725	5.56
Cherif_Lo	10	123	17	5043	40.84
Thienaba	10	172	35	3807	34.49
Ndieyene_Shirak	11	288	51	6377	62.43
Notto_Gouye_Diama	11	20	3	2491	12.72
Malicounda_Wolof	12	16	1	2190	11.7
Mbayene	12	305	59	3728	39.78
Pekesse	12	154	15	5669	46.5
Thiadiaye	13	268	59	5460	40.66
Thilmanka	14	25	1	2445	20.35
Mont_Roland	15	808	169	7302	106.04
Sandira	15	59	13	2846	29.49
Koul	16	408	79	6350	108.44
Meouane	17	277	51	5350	83.05
Neugeniene	17	182	23	4886	81.65
Ndiass	18	332	87	5734	88.5
Pire_Goureye	18	559	113	6854	138.85
Merina_Dakhar	19	448	91	4683	85.16
Ngandiouf	19	660	139	4171	129.67
Nguekhokh	19	717	199	6110	121.03
Touba_Toul	20	1845	397	8545	292.24
Tassette	21	305	51	7060	154.45
Diender_Guedj	22	1714	385	10891	389.12
Ndiagagniao	24	3906	915	11495	793.63
Notto	28	13451	3429	13171	2019.97
Pout	29	7246	1965	15670	1159.21

## Algorithm: lower bound set generation

Algorithm 2 describes lower bound set generation at any given node of the branch-and-bound tree. It is based on the algorithm by Aneja and Nair (1979). Input parameter *node* represents the set of branching decisions that define this node, while *UB* is the upper bound set. Calls to *solve*() involve calling a mixed-integer linear programming solver, either on a lexmin function or using a weighted sum, and generating cuts in the process. *UB* is passed as parameter to *solve*() since the upper bound set is updated every time a new integer solution is encountered. *C* is the collection of pairs of points that still need to be investigated, while *E* is the set of *segments* that have each been proved to define a linear portion of the LB set. Functions *push*()/*pop*() operate in the traditional way and add/retrieve an element to/from a collection, respectively. Coordinates of a point are accessed using subscript indices, e.g., $p = (p_1, p_2)$.

For more details and the complete version of this algorithm, we refer to Parragh and Tricoire (2019).
